# Carbon quantum dots of ginsenoside Rb1 for application in a mouse model of intracerebral Hemorrhage

**DOI:** 10.1186/s12951-024-02368-w

**Published:** 2024-03-22

**Authors:** Xiaolong Tang, Xinyu Yang, Yamei Yu, Miaojing Wu, Yuanyuan Li, Zhe Zhang, Guangyu Jia, Qi Wang, Wei Tu, Ye Wang, Xingen Zhu, Shiyong Li

**Affiliations:** 1 Department of Neurosurgery, The Second Affiliated Hospital, Jiangxi Medical College, Nanchang University, Nanchang, Jiangxi 330006 China; 2 Institute of Neuroscience, Nanchang University, Nanchang, Jiangxi 330006 China; 3 Department of Neurology, The Second Affiliated Hospital, Jiangxi Medical College, Nanchang University, Nanchang, Jiangxi 330006 China

**Keywords:** Carbon quantum dots, Ginsenoside Rb1, Reactive oxygen species, iron overload, Oxidative stress

## Abstract

After intracerebral hemorrhage (ICH) occurs, the overproduction of reactive oxygen species (ROS) and iron ion overload are the leading causes of secondary damage. Removing excess iron ions and ROS in the meningeal system can effectively alleviate the secondary damage after ICH. This study synthesized ginsenoside Rb1 carbon quantum dots (RBCQDs) using ginsenoside Rb1 and ethylenediamine via a hydrothermal method. RBCQDs exhibit potent capabilities in scavenging ABTS + free radicals and iron ions in solution. After intrathecal injection, the distribution of RBCQDs is predominantly localized in the subarachnoid space. RBCQDs can eliminate ROS and chelate iron ions within the meningeal system. Treatment with RBCQDs significantly improves blood flow in the meningeal system, effectively protecting dying neurons, improving neurological function, and providing a new therapeutic approach for the clinical treatment of ICH.

**Supplementary Information:**

The online version contains supplementary material available at 10.1186/s12951-024-02368-w.

## Introduction

ICH is a severe form of hemorrhagic brain injury associated with high rates of disability and mortality [1–3]. ROS and iron ion overload are the primary contributors to the secondary damage following ICH. After ICH, red blood cells rapidly infiltrate the subarachnoid space of the meningeal system. The rupture of these red blood cells leads to the generation of substantial amounts of hemoglobin, free iron ions, ROS, and inflammatory mediators. These toxic substances can potentially impair the meningeal lymphatic system, obstructing the clearance of metabolic waste within the brain. In turn, the dysfunction of the meningeal lymphatic system results in the further accumulation of free iron ions and ROS in brain tissue, thereby exacerbating the severity of inflammation and neural damage. Consequently, the clearance of excess iron ions and ROS from the meningeal lymphatic system emerges as an effective strategy for mitigating secondary brain damage.

The intrathecal injection delivers drugs directly into the cerebrospinal fluid, allowing drugs to reach the subarachnoid space and the meningeal lymphatic system directly. This approach provides an effective drug delivery method for clearing ROS and iron ions from the meningeal lymphatic system. Deferoxamine (DFO) [4]is commonly used to remove excess iron ions. However, due to its singular target specificity, DFO cannot eliminate ROS, limiting its effectiveness in treating ICH. Following ICH, antioxidants such as vitamin C [5], thiol-based drugs [6], and enzymatic antioxidants [7] can alleviate cellular damage caused by oxidative stress. Nevertheless, these agents are insufficient for clearing free iron ions. Therefore, there is an urgent need to develop a multifunctional nanomedicine capable of effectively removing excessively released iron ions and eliminating ROS for the clinical treatment of hemorrhagic brain injury.

**Fig. 1:**
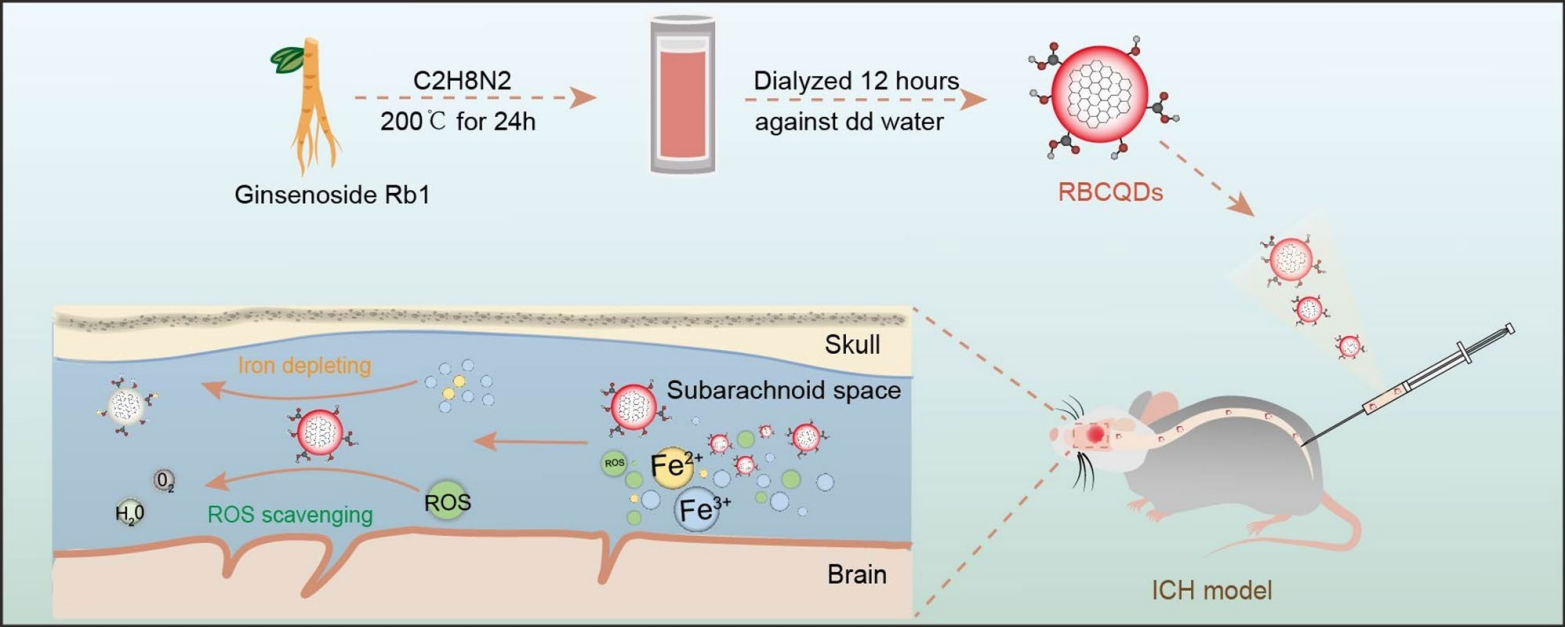
Schematic representation of RBCQDs synthesis and their application in intracerebral hemorrhage treatment

Carbon quantum dots (CQDs), referring to carbon quantum dots with fluorescent properties and sizes smaller than 10 nm, typically consist of a hybrid carbon core and a modified functional group shell [8]. These particles have been widely applied in the treatment of neurological disorders. CQDs have shown effectiveness in enhancing memory and learning in Alzheimer’s Disease (AD) treatment and in preserving myelin integrity while reducing glial scarring in traumatic spinal cord injuries [9, 10]. Ginsenoside Rb1, the key active compound in Panax ginseng, is noted for its anti-inflammatory, antioxidant, and anti-apoptotic effects [11]. However, its limited solubility and blood-brain barrier penetration hinder its clinical effectiveness [12]. In our study, to address the clearance of iron ions and ROS, we utilized ginsenoside Rb1 and ethylenediamine as raw materials to synthesize a type of CQDs named RBCQDs via a hydrothermal method. Our data indicates that the size of RBCQDs ranges from 6 to 8 nm, demonstrating effective capability in clearing free iron ions and ROS. Intrathecal injection of RBCQDs for treating ICH removes the excess accumulation of ROS and iron ions within the meningeal system, leading to improved recovery of neurological function in mice with ICH. This research holds promise for offering novel therapeutic approaches in the treatment of hemorrhagic stroke (Fig. 1). 

## Results and discussions

### Characterization of RBCQDs

**Fig. 2:**
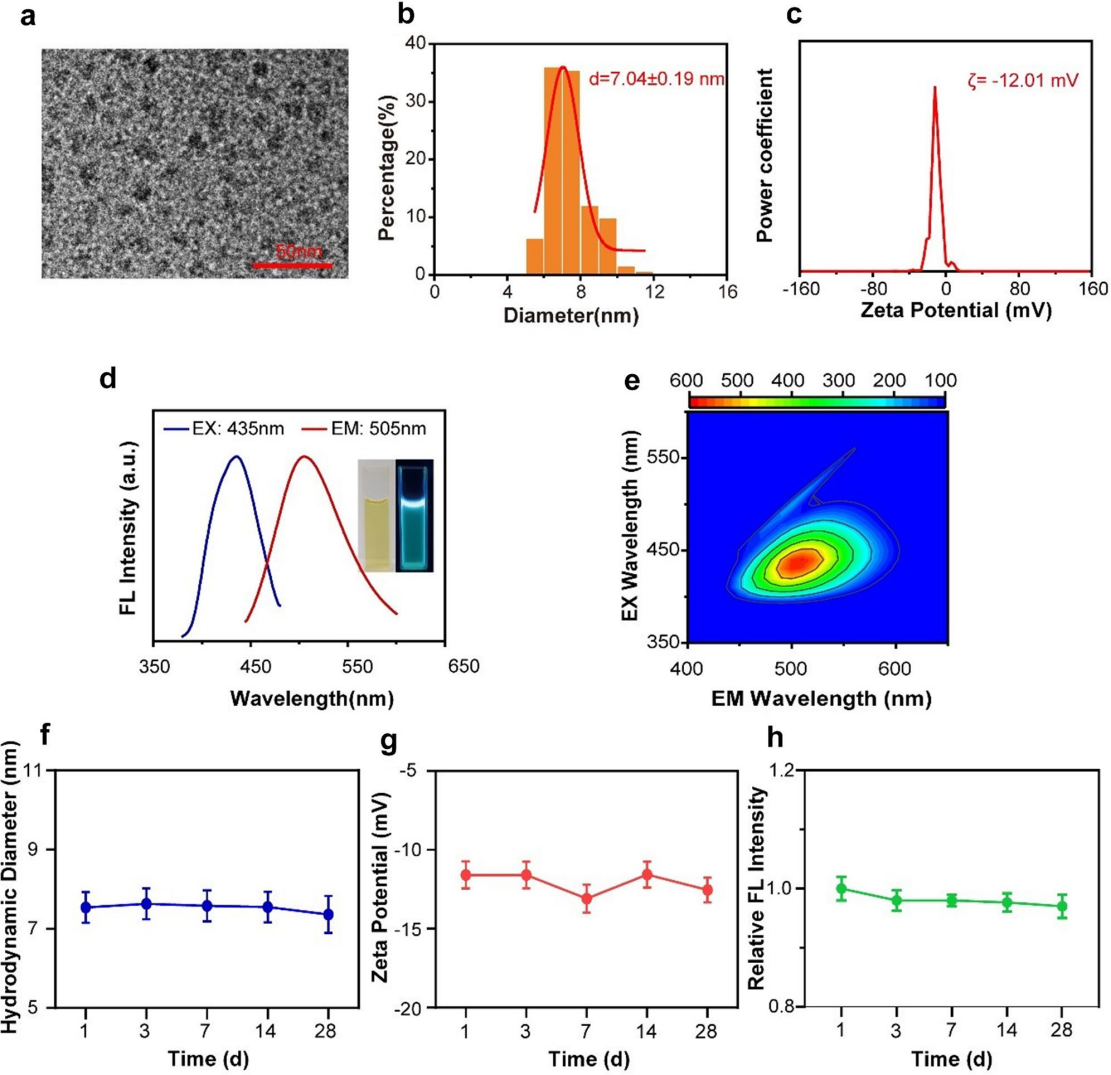
Characterization of RBCQDs. (**a**) TEM image of RBCQDs. (**b**) The hydrodynamic diameter of RBCQDs is measured by dynamic light scattering. (**c**) Zeta potential of RBCQDs. (**d**) Optimal excitation (blue line, *λ* ex = 435 nm) and emission spectra (red line, *λ* em = 505 nm) of RBCQDs, Inset images on the right, photographs of RBCQDs under brightfield and 365 nm UV illumination. (**e**) 3D fluorescence spectra of RBCQDs. Changes in size (**f**), zeta potential (**g**) and fluorescence intensity (**h**) of RBCQDs over time in artificial cerebrospinal fluid (*n* = 6/group, one-way ANOVA)

This study successfully synthesized RBCQDs using ginsenoside Rb1 and ethylenediamine through a hydrothermal method at 200 °C. Transmission electron microscopy (TEM) analysis revealed that the synthesized RBCQDs exhibited a quasi-spherical structure with good dispersibility, with an average diameter of approximately 6–10 nm (Fig. 2a). High-resolution transmission electron microscopy (HRTEM) images (Fig. S1a) revealed a clear crystalline structure of RBCQDs with an interplanar spacing of about 0.21 nm, matching the (100) lattice plane of graphene, indicating that RBCQDs are primarily composed of sp^2^-hybridized carbon atoms [13]. As shown in Fig. 2b, the hydrodynamic diameter of RBCQDs in artificial cerebrospinal fluid was 7.04 ± 0.19 nm, consistent with the physical diameter observed by TEM. The Zeta potential of RBCQDs was approximately − 12.04 ± 0.85 mV, indicating a negatively charged surface (Fig. 2c).

Additionally, RBCQDs exhibited excellent optical properties. As depicted in Fig. 2d, the optimal excitation wavelength for RBCQDs was 435 nm, with an emission wavelength of 505 nm, emitting bright cyan fluorescence under 365 nm UV illumination. To investigate the fluorescence properties of RBCQDs further, we examined their 3D fluorescence emission spectra at different excitation wavelengths from 350 to 600 nm (Fig. 2e). The results indicated that the emission wavelengths of RBCQDs were mainly concentrated in the range of 490 to 550 nm, and the fluorescence intensity showed an increasing and then decreasing trend with the excitation wavelength, potentially due to the quantum size effect and the diversity of luminescent sites on the surface of RBCQDs [14]. Furthermore, we conducted a long-term observation of the physical stability of RBCQDs in artificial cerebrospinal fluid (Fig. 2f-h). The results demonstrated that the size, zeta potential, and fluorescence intensity of RBCQDs showed no significant changes during the 28-day observation period, confirming their excellent physical stability.

## Clearance of oxygen free radicals and iron ions by RBCQDs in solution

**Fig. 3:**
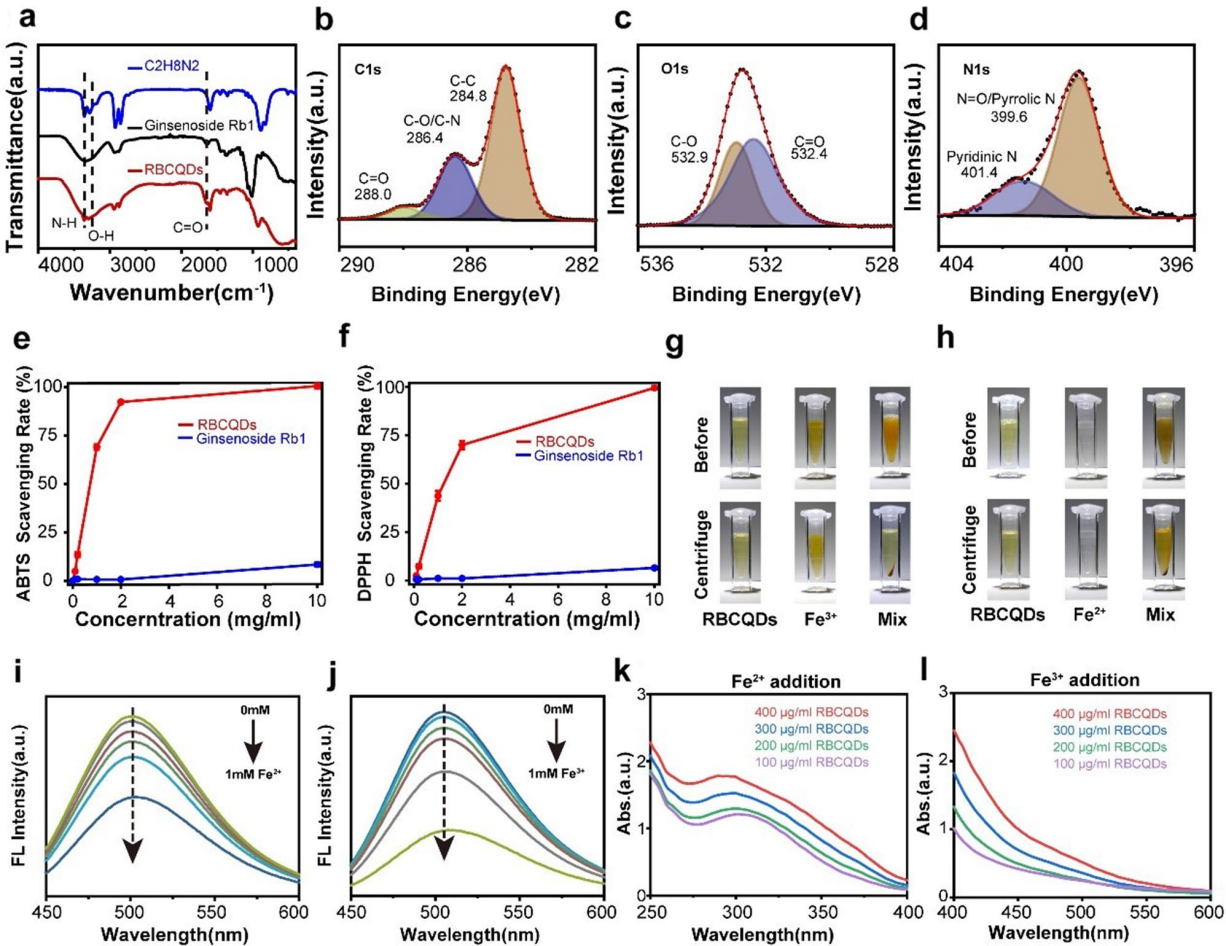
Effective clearance of oxygen free radicals and iron ions by RBCQDs. (**a**) FT-IR spectra of ethylenediamine (blue), ginsenoside Rb1 (black), and RBCQDs (red). XPS spectra of RBCQDs, including C1s (**b**), O1s (**c**), and N1s (**d**) exemplary spectra. The clearance rates of ABTS (**e**) and DPPH (**f**) radicals by RBCQDs and ginsenoside Rb1 at various concentrations (*n* = 6/group, two-way ANOVA). (**g**) Appearance before and after RBCQDs, Fe^3+^, and RBCQDs + Fe^3+^ centrifugation. (**h**) Appearance before and after centrifugation of RBCQDs, Fe^2+^, and RBCQDs + Fe^2+^. (**i**) Fluorescence intensity of RBCQDs after incubation with 0, 0.05, 0.15, 0.25, 0.5, and 1 mM Fe^2+^. (**j**) Fluorescence intensity of RBCQDs after incubation with 0, 0.05, 0.15, 0.25, 0.5, and 1 mM Fe^3+^. (**k**) UV absorption spectra of RBCQDs at different concentrations after incubation with Fe^2+^ for 12 h. (**l**) UV absorption spectra of RBCQDs at different concentrations after incubation with Fe^3+^ for 12 h

Previous studies have demonstrated a close correlation between the antioxidant properties and iron-chelating ability of quantum dots, their sp²-hybridized carbon cluster structure, and surface functional groups such as hydroxyl, carbonyl, and amino groups [15–17]. We detailedly characterized the surface functional groups and elemental composition of ginsenoside Rb1 and RBCQDs using Fourier transform infrared spectroscopy (FTIR) and X-ray photoelectron spectroscopy (XPS). FTIR analysis results (Fig. 3a) showed absorption peaks of RBCQDs at 3361 cm^-1^ corresponding to N-H vibration [18], 3332 cm^-1^ related to O-H vibration [19], and the absorption peak at 1651 cm^-1^ indicates the presence of C = O bonds [19]. These features indicate that RBCQDs retained the main chemical structure of ginsenoside Rb1 and successfully introduced amino modification, enhancing their interaction capability with metal ions. XPS analysis further revealed the elemental composition and chemical structure of RBCQDs and ginsenoside Rb1. Apart from hydrogen, the percentages of carbon, oxygen, and nitrogen elements in RBCQDs were 75.23%, 19.78%, and 4.99%, respectively (Fig. S1b). Compared to ginsenoside Rb1 (Fig. S1c-e), RBCQDs exhibited a higher carbon content, possibly related to the carbonization process of the raw materials. In the C1s spectrum analysis of RBCQDs (Fig. 3b), three types of peaks were fitted, corresponding to C-C (284.8 eV), C-O/C-N (286.4 eV), and C = O (288.0 eV) [20]. The O1s spectrum analysis (Fig. 3c) resolved into two peaks at 532.4 and 532.9 eV, corresponding to C = O and C-O [21]. The N1s spectrum presented two components at 399.6 and 401.4 eV (Fig. 3d), corresponding to N = O/pyrrolic N and pyridinic N [22]. These characterizations suggest that RBCQDs are mainly composed of sp²-hybridized carbon clusters. Compared to ginsenoside Rb1, RBCQDs have a higher carbon content and underwent amino modification, potentially enhancing their ability to clear oxygen free radicals and iron ions.

To compare the antioxidant capacity between ginsenoside Rb1 and RBCQDs, we performed detection using ABTS + and DPPH• reagent kits (Fig. 3e, f). ABTS cation and DPPH radicals typically appear blue and purple in solution. Reaction with antioxidant substances leads to a reduction in absorbance, allowing the quantification of the antioxidant capacity. Experimental results indicated that RBCQDs significantly outperformed ginsenoside Rb1 in clearing ABTS cation and DPPH radicals.

Furthermore, we assessed the iron chelating ability of ginsenoside Rb1 and RBCQDs. As shown in Fig. S2a, b and Fig. 3g, h, we added Fe^2+^ or Fe^3+^ separately to ginsenoside Rb1 and RBCQDs solutions. The results demonstrated that the mixed solution of RBCQDs quickly changed from clear to turbid, while the solution of ginsenoside Rb1 showed no significant change. After co-incubation of RBCQDs and Fe^2+^ or Fe^3+^ for 12 h and subsequent centrifugation, distinct brown or reddish-brown chelation products appeared at the bottom of the centrifuge tube, indicating a chelation reaction between RBCQDs and Fe^2+^ or Fe^3+^. With an increase in the concentration of Fe^2+^ or Fe^3+^ added to the RBCQDs solution, not only did the solution’s transparency gradually decrease, but the blue light intensity emitted by the solution under 365 nm ultraviolet irradiation also gradually weakened (Fig. S2c, d). To quantitatively analyze the chelating ability of RBCQDs with Fe^2+^ and Fe^3+^, we conducted Stern-Volmer fluorescence quenching experiments to analyze the chelating ability of RBCQDs with Fe^2+^ and Fe^3+^ [23]. Experimental data showed that the fluorescence intensity of RBCQDs increased linearly with the concentration of iron ions in the solution within the range of 0-0.5 mM (Fig. 3i, j and Fig. S3). Finally, we analyzed the chelating ability of ginsenoside Rb1 and RBCQDs with Fe^2+^ and Fe^3+^ using ultraviolet visible (UV-vis) spectroscopy (Fig. 3k, l and Fig. S4, 5). The results indicated that, compared to the sole RBCQDs solution, the mixed solution of RBCQDs and Fe^2+^ or Fe^3+^ exhibited concentration-dependent UV absorption peak enhancements at 250–400 nm or 400–600 nm, suggesting the formation of chelation products between RBCQDs and Fe^2+^ or Fe^3+^. In contrast, no significant UV absorption changes were observed in the mixed solution of ginsenoside Rb1 and Fe^2+^ or Fe^3+^ in the 220–1000 nm wavelength range, indicating its inability to chelate Fe^2+^ or Fe^3+^ ions. In summary, consistent with the characterization results of ginsenoside Rb1 and RBCQDs, RBCQDs exhibit significant antioxidant and iron chelating capabilities in solution compared to ginsenoside Rb1.

**Fig. 4:**
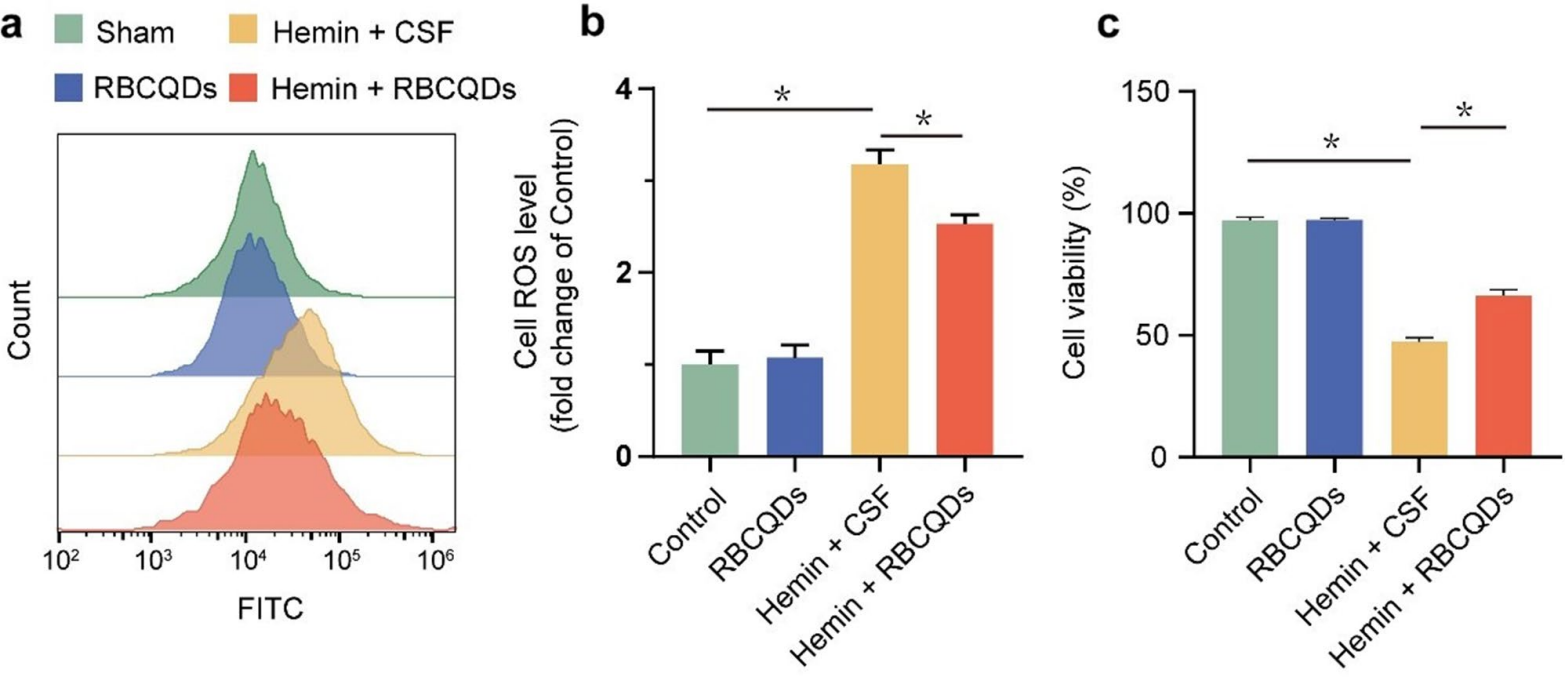
Alleviation of oxidative stress damage in HT22 cells in the hemin microenvironment by RBCQDs. Quantifying ROS labeled with DCFH-DA probe in HT22 cells using flow cytometry (**a**) and corresponding results (**b**) (*n* = 6/group, one-way ANOVA). (**c**) Cell viability of HT22 cells assessed by the CCK-8 assay (*n* = 6/group, one-way ANOVA)

### Mitigation of oxidative stress damage in HT22 cells by RBCQDs

We further validated the antioxidant capacity of RBCQDs by examining their impact on HT22 cells in the hemin microenvironment. Hemoglobin degradation products, such as Hemin, are primary toxic metabolites that induce oxidative stress damage and cellular toxicity in vitro [24]. We utilized flow cytometry to assess changes in intracellular ROS levels in HT22 cells. The results revealed a significant increase in ROS levels in HT22 cells treated with Hemin compared to the control group. However, treatment with RBCQDs led to a notable reduction in ROS levels in HT22 cells (Fig. 4a, b). The CCK-8 assay evaluated the viability of HT22 cells exposed to the hemin environment. The findings demonstrated a substantial decrease in cell viability under hemin induction. In contrast, intervention with RBCQDs significantly alleviated the hemin-induced decline in cell viability (Fig. 4c). In summary, RBCQDs exhibit the ability to effectively eliminate intracellular ROS, providing cellular protection against hemin-induced oxidative stress.

### Distribution of RBCQDs in the central nervous system following intrathecal injection

**Fig. 5:**
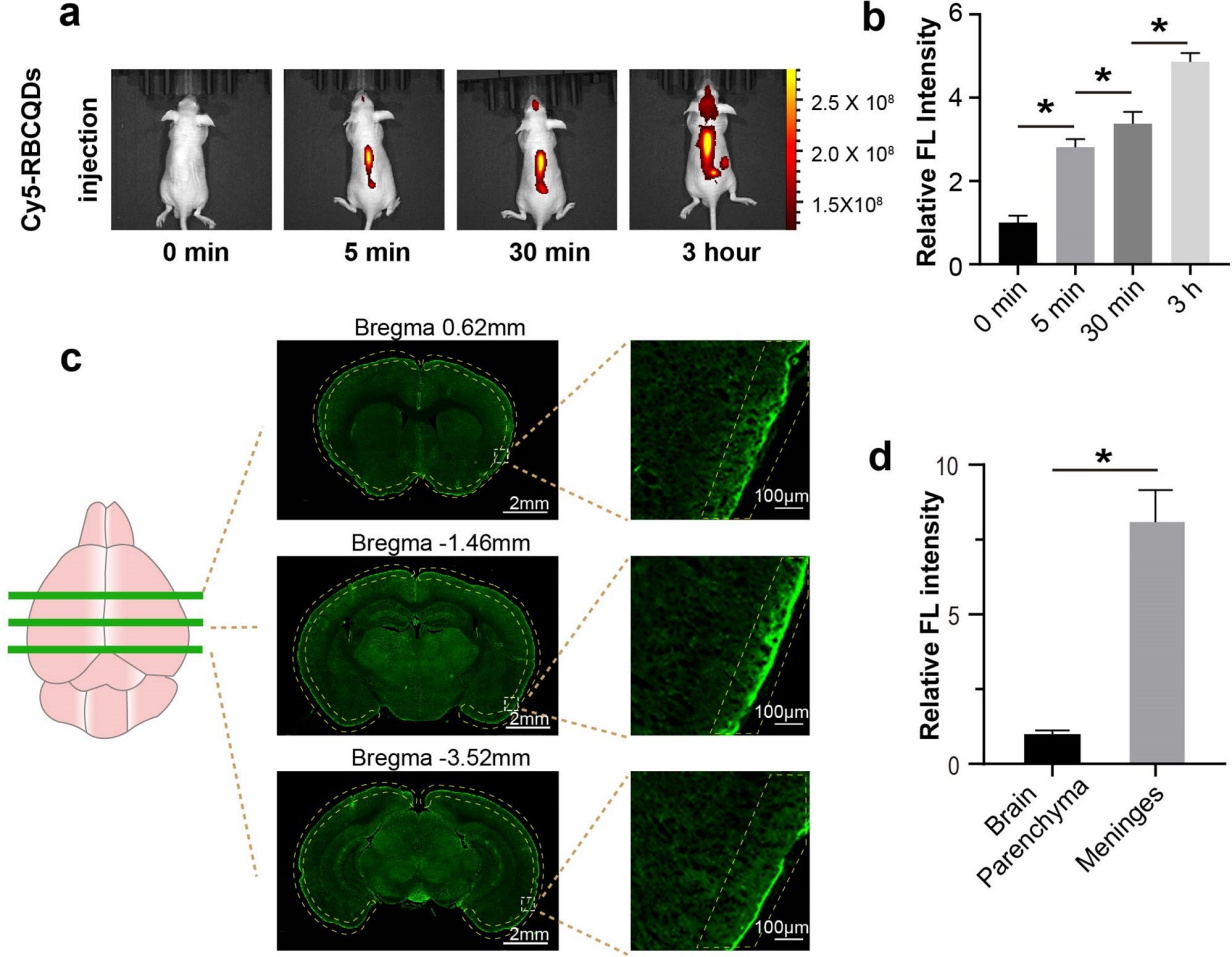
Distribution of RBCQDs in the central nervous system. (**a**) Spatial distribution of Cy5-RBCQDs in the intracranial space at 0 min, 5 min, 30 min, and 3 h post intrathecal injection, and (**b**) corresponding relative fluorescence intensity (*n* = 6/group, one-way ANOVA). (**c**) Distribution of RBCQDs in the brain 30 min after intrathecal injection and (**d**) relative fluorescence intensity in the meninges (*n* = 6/group, paired t-test)

Intrathecal administration is a method of drug delivery involving the direct injection of a substance into the cerebrospinal fluid, bypassing the blood brain barrier [25]. This approach allows drug molecules to act directly on the central nervous system, potentially enhancing therapeutic efficacy. Utilizing a small animal in vivo imaging system, we investigated the distribution and temporal dynamics of Cy5-labeled RBCQDs in the central nervous system. From 5 min to 3 h post intrathecal injection, RBCQDs predominantly accumulated in the spinal cord and cranial regions. The increasing quantity of RBCQDs in the brain over time suggests their penetration into the subarachnoid space of the cranial compartment (Fig. 5a, b). Furthermore, we harvested brain tissue 30 min after intrathecal injection of RBCQDs and observed their distribution using the fluorescent properties of RBCQDs. A stronger signal of RBCQDs was evident on the meninges compared to the brain parenchyma, indicating the primary accumulation of RBCQDs in the subarachnoid or on the brain surface (Fig. 5c, d).

### Clearance of the peri-hemorrhagic brain tissue microenvironment by RBCQDs

**Fig. 6:**
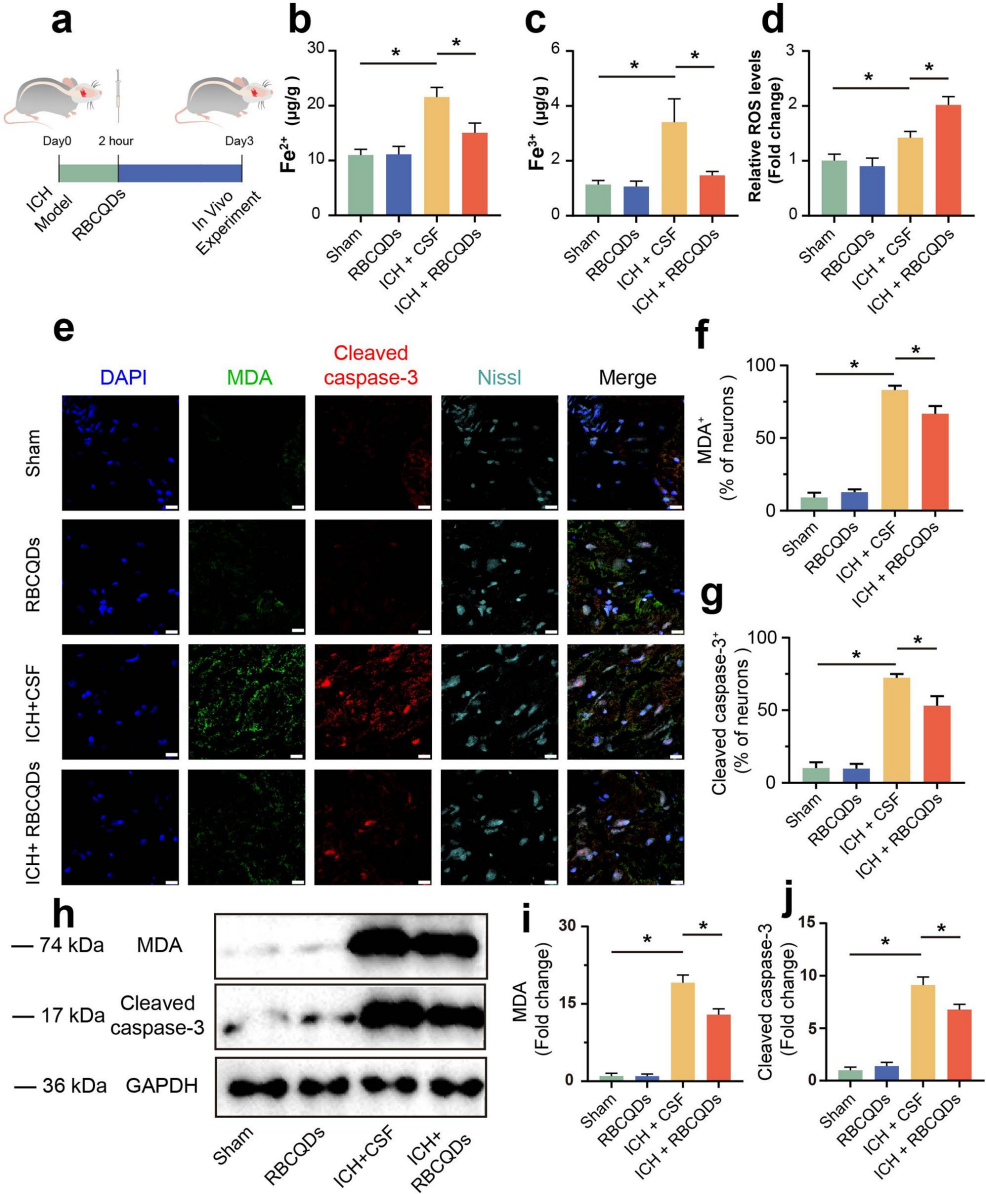
RBCQDs reduce brain tissue iron content and alleviate oxidative stress damage after ICH. (**a**) Experimental timeline for in vivo mouse studies. Colorimetric assay to detect Fe^2+^ (**b**) and Fe^3+^ (**c**) levels in brain tissue (*n* = 6/group, one-way ANOVA). (**d**) DCFH- DA probe assay to measure ROS levels in mouse brain tissue cells (*n* = 6/group, one-way ANOVA). (**e**) Immunofluorescence staining images showing the content of MDA and cleaved caspase-3 in neurons of brain tissue. Nissl staining marks neurons, FITC labels MDA, TRITC labels cleaved caspase-3, and DAPI marks cell nuclei.The scale bar length in (**e**) was 20 μm. Relative fluorescence intensity of MDA (**f**) and cleaved caspase-3 (**g**) in neurons of brain tissue (*n* = 6/group, one-way ANOVA). Immunoblot bands (**h**) and quantitative analysis showing the content of MDA (**i**) and cleaved caspase-3 (**j**) in neurons of brain tissue in different treatment groups (*n* = 6/group, one-way ANOVA)

After ICH, hemoglobin present in brain tissue degrades to produce iron and ROS, leading to iron overload and oxidative stress damage in the central nervous system [24]. To observe the impact of RBCQDs on iron deposition and oxidative stress response in mouse brain tissue after ICH, we established a mouse collagenase induced ICH model and administered RBCQDs intrathecally 2 h after intracerebral injection of collagenase (Fig. 6a). Colorimetric assays revealed that RBCQDs significantly inhibited the deposition of Fe^2+^ and Fe^3+^ ions in brain tissue after ICH, alleviating iron overload (Fig. 6b, c). Additionally, RBCQDs markedly reduced ROS levels in brain tissue cells after ICH, mitigating oxidative stress levels (Fig. 6d).

Excessive production of oxygen free radicals after ICH can lead to oxidative stress damage and activate multiple signaling pathways, inducing and exacerbating neuronal apoptosis. Malondialdehyde (MDA) is the final product of lipid peroxidation in neurons and is known to bind to proteins and nucleic acids, among others exhibiting cellular toxicity. It is commonly used as an indicator of tissue oxidative stress severity [26]. Caspase-3 is an effector molecule in the apoptotic pathway, and activated caspase-3 (cleaved caspase-3) is an essential marker for assessing the apoptosis process [27]. Our immunofluorescence results demonstrated (Fig. 6e-g) that, compared to the Sham group, the content of MDA and cleaved caspase-3 in neurons significantly increased after ICH. Following treatment with RBCQDs, MDA and cleaved caspase-3 content in neurons markedly decreased. Western blot results further confirmed this phenomenon (Fig. 6h-j), indicating that RBCQDs effectively reduce the expression of MDA and cleaved caspase-3 proteins in brain tissue after ICH. These results suggest that RBCQDs significantly alleviate oxidative stress damage and reduce neuronal apoptosis in brain tissue after ICH, revealing their potential neuroprotective mechanism.

**Fig. 7:**
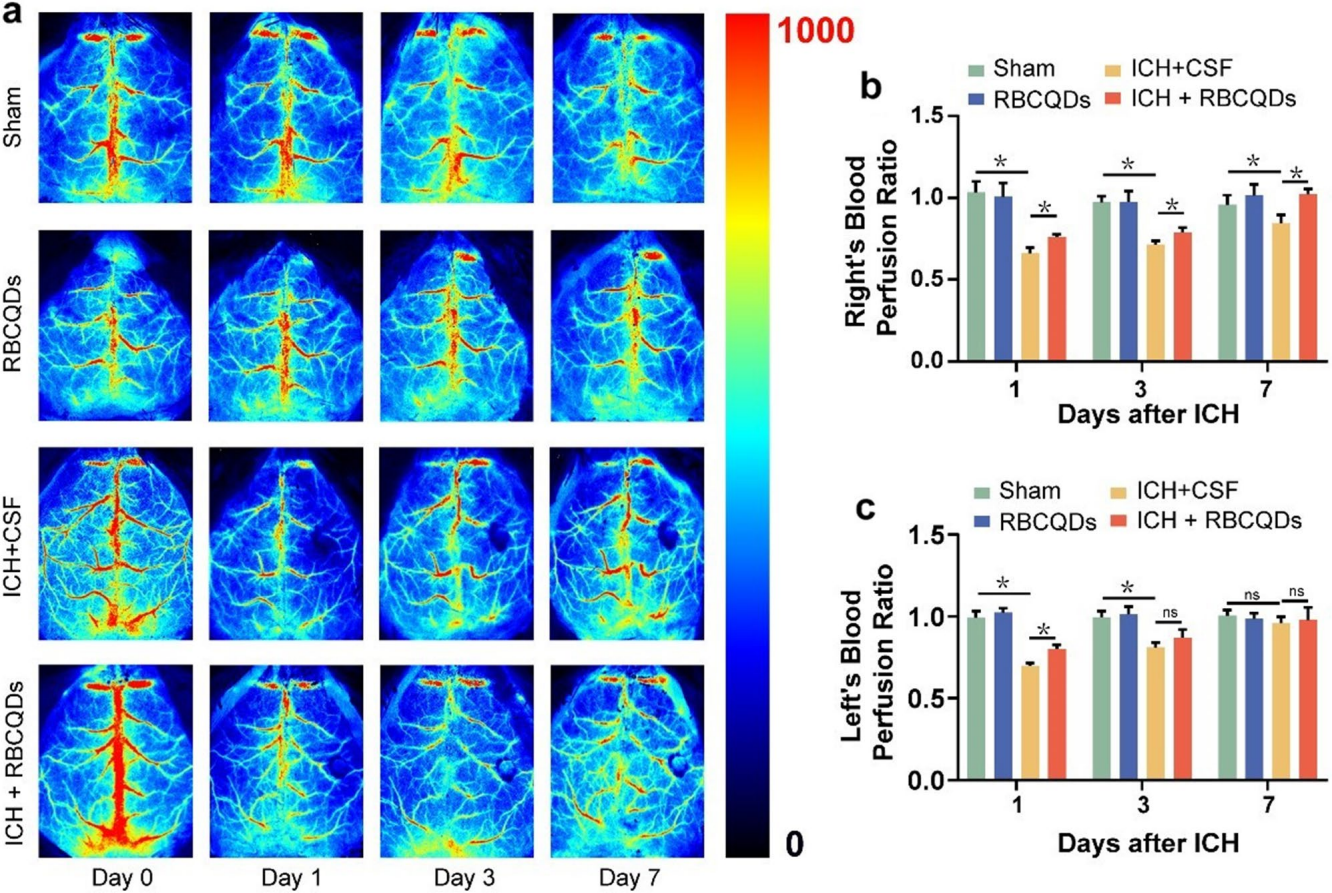
RBCQDs promote post-ICH recovery of cerebral dura mater blood perfusion in mice. (**a**) LSI images depicting the dura mater perfusion status at different time points for Sham, RBCQDs, ICH + CSF, and ICH + RBCQDs groups. Relative perfusion levels in the dura mater on the right side (**b**) and left side (**c**) of mice across different treatment groups and time points (*n* = 6/group, two-way ANOVA)

### RBCQDs improve cerebral dura mater blood perfusion on both sides

The meningeal system plays a crucial role in the clearance of metabolic waste from the central nervous system. Following cerebral hemorrhage, functional impairment of the meningeal system obstructs the elimination of metabolic waste, contributing significantly to the worsening of secondary damage after cerebral hemorrhage [28–31]. We utilized laser speckle imaging (LSI) to investigate the impact of RBCQDs on the average perfusion of the mouse dural vasculature system. As depicted in Fig. 7a-c, post-ICH, there was a notable reduction in blood perfusion in both the right and left dura mater regions. However, treatment with RBCQDs significantly increased blood perfusion in the right dura mater region on days 1, 3, and 7 post-ICH and in the left dura mater region on day 1. This enhancement facilitates the clearance of metabolic waste through the dural route, alleviating cerebral tissue damage after hemorrhagic events.

### Neurological functional assessment in mice

**Fig. 8:**
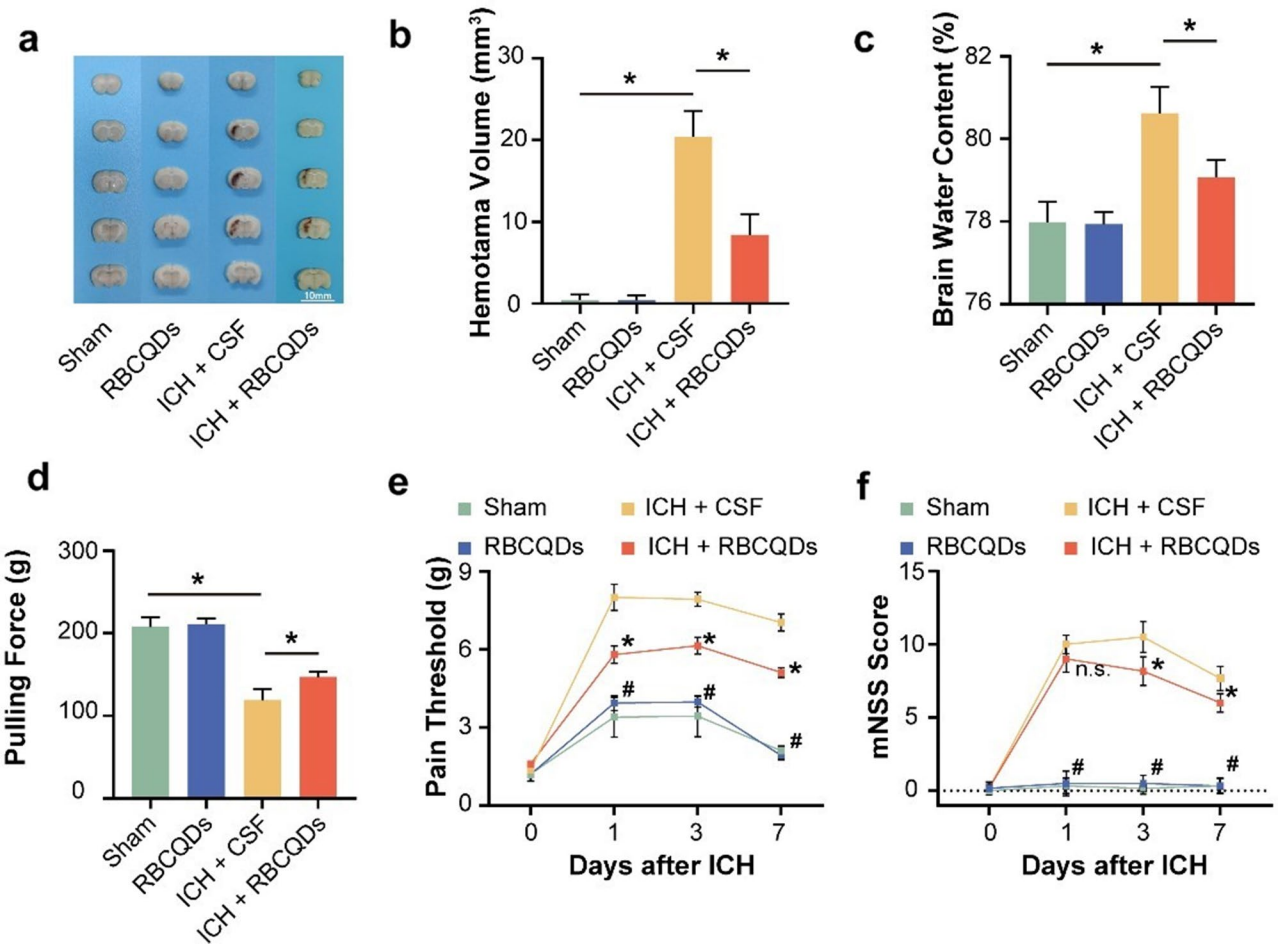
RBCQDs reduce brain hemorrhage volume and brain water content, Promoting Neurological Function Recovery. Images (**a**) and quantitative analysis (**b**) of brain hematomas for mice from the Sham, RBCQDs, ICH + CSF, and ICH + RBCQDs groups are presented three days following ICH (*n* = 6/group, one-way ANOVA). (**c**) Brain water content is also shown on the 3 days after ICH in Sham, RBCQDs, ICH + CSF, and ICH + RBCQDs groups (*n* = 6/group, one-way ANOVA). (**d**) Limb strength is shown on the 3 day after ICH in the Sham, RBCQDs, ICH + CSF, and ICH + RBCQDs groups (*n* = 6/group, one-way ANOVA). Paw pain threshold (**e**) and mNSS (**f**) of mice before ICH, 1 day after ICH, 3 days after ICH, and 7 days after ICH in Sham, RBCQDs, ICH + CSF, and ICH + RBCQDs groups (*n* = 6/group, two-way ANOVA). In Fig. 8e, f, “*” and “#” both indicate *p* < 0.05, “*” for sham vs. ICH + CSF and “#” for ICH + CSF vs. ICH + RBCQDs

We further investigated the role of RBCQDs treatment on ICH hematoma volume and brain water content. The results showed that, compared with the Sham group, the ICH + CSF group exhibited a significant increase in brain tissue water content and hematoma volume. However, intrathecal administration of RBCQDs significantly alleviated these changes induced by ICH (Fig. 8a-c). Intracerebral hematoma and brain edema not only cause increased intracranial pressure by compressing brain tissue but also directly lead to brain tissue damage, increasing the risk of disability and mortality [32, 33]. RBCQDs effectively reduced hematoma volume and brain tissue water content, potentially contributing to the recovery of neurological function in mice.

Secondly, we objectively assessed the severity of nerve damage in mice by measuring forelimb grip strength, left hind limb pain threshold, and modified neurological severity score (mNSS). In grip strength measurement, we observed that ICH mice treated with RBCQDs exhibited significantly improved grip strength on the 7 days compared to the ICH + CSF group, indicating that RBCQDs promote the recovery of limb strength in ICH mice (Fig. 8d). In the pain threshold experiment, ICH + RBCQDs mice showed a significantly lower pain threshold on the 1, 3, and 7 days compared to the ICH + CSF group, indicating a positive effect of RBCQDs on sensory function recovery (Fig. 8e). As shown in Fig. 8f, RBCQDs significantly reduced the mNSS scores of ICH mice on the 3 and 7 days, suggesting a significant improvement in the overall neurological function status.

Finally, we assessed the clearance and the potential toxicity of the RBCQDs. As illustrated in Fig. S6, a detectable quantity of RBCQDs persists within the cerebral environment 3 days following intrathecal administration, establishing a robust basis for sustained therapeutic efficacy. The HE staining results (Fig. S7) reveal no significant pathological alterations in the organs of mice treated with RBCQDs, and weight monitoring (Fig. S8a, b) indicates comparable weight dynamics across Sham and RBCQD groups, as well as ICH + CSF and ICH + RBCQD groups, collectively suggesting the superior biocompatibility of RBCQDs.

RBCQDs showcase strong potential in treating cerebral hemorrhage due to their excellent water solubility, antioxidative, and iron chelation capabilities. They not only alleviate secondary brain damage post-injury but also promote neurological recovery. Moreover, their metal-free composition ensures outstanding biocompatibility, with no significant organ damage, proving their safety and feasibility for clinical use. Thus, RBCQDs hold significant clinical translation value for cerebral hemorrhage treatment and functional recovery, meriting further research and development.

## Conclusion

RBCQDs, with antioxidant and iron chelating properties, demonstrate potential as novel therapeutic agents for ICH. In comparison to ginsenoside Rb1, RBCQDs not only demonstrate superior water solubility and antioxidative efficiency but also exhibit a remarkable capacity for iron chelation, thereby enhancing their effectiveness in mitigating secondary damage following cerebral hemorrhage. Applying RBCQDs in ICH mice reduced iron ion levels and ROS content in the tissues surrounding the hematoma. Furthermore, using RBCQDs reduced hematoma volume and edema content, alleviating secondary damage from cerebral hemorrhage and promoting the protection of brain neurofunction. These findings provide new nanodrugs for ICH treatment.

## Materials and methods

### Synthesis of RBCQDs

RBCQDs were synthesized through a one-step hydrothermal method. In brief, 1 g of ginsenoside Rb1 was thoroughly mixed with 3 ml of anhydrous ethylenediamine in 10 ml of double-distilled water and stirred. The mixture was then transferred to a high-pressure reaction kettle with a polytetrafluoroethylene inner liner and subjected to hydrothermal synthesis at 200 °C for 24 h. The resulting solution was cooled to room temperature, and large particle impurities were removed by filtration through a 0.22 μm microporous membrane, yielding a brown transparent solution. In a light-avoiding environment, the solution underwent 12 h of dialysis purification using a membrane with a molecular weight cutoff of 1 kDa. The purified RBCQDs solution was then freeze-dried for storage.

### Characterization of RBCQDs

High resolution transmission electron microscopy (Talos F200S G2, Thermo Scientific) was employed to analyze the morphology and size of RBCQDs with an acceleration voltage set at 200 kV. Fourier transform infrared spectrometry (Nicolet iS50, Thermo Scientific) was used to investigate the infrared absorption spectra of ginsenoside Rb1, anhydrous ethylenediamine, and RBCQDs, with samples prepared using a potassium bromide pellet method. X-ray photoelectron spectrometry (Nexsa G2, Thermo Scientific) was utilized to analyze ginsenoside Rb1 and RBCQDs with a 300 W Al K radiation source. The fluorescence spectrometer (F-4700, HITACHI) was employed to evaluate the 3D fluorescence characteristics of RBCQDs. NanoBrook 90Plus PALS (BROOKHAVEN) was used for determining the hydrodynamic diameter and potential of RBCQDs.

### Preparation of Cy5-RBCQDs fluorescent probe

Following the methodology outlined in reference [34], the fluorescent probe Cy5-RBCQDs was synthesized by esterification reaction, coupling the hydroxyl groups of RBCQDs with the carboxyl groups on Cy5. In brief, RBCQDs and Cy5 were dissolved in N, N-dimethylformamide (DMF) solvent, thoroughly mixed, and 4-dimethylaminopyridine (DMAP) was added to the mixture. After reacting at room temperature for 24 h, 1,3-dicyclohexylcarbodiimide (DCC) was introduced, and the reaction continued in the dark for another 24 h. The reaction mixture was then poured into anhydrous ether, causing the product to precipitate, which was subsequently purified.

### ABTS + clearance experiment

Following the manufacturer’s instructions, the liquid sample total antioxidant capacity Assay Kit (E2006, APPLYGEN) was utilized to assess the scavenging capacity of ginsenoside Rb1 and RBCQDs on free radicals. Solutions of ginsenoside Rb1 and RBCQDs with concentrations of 0, 0.1, 0.2, 1, 2, and 10 mg/mL were added to the ABTS + solution, thoroughly mixed, and then incubated at room temperature for 5 min. The absorbance of the solution was measured at 734 nm (A1). Absorbance values were obtained by replacing the sample with PBS (A0).

$${\rm{ABTS}}\,{\rm{Radical}}\,{\rm{Scavenging}}\,{\rm{Rate}}\left( \% \right) = \left[ {\left( {{\rm{A}}0 - {\rm{A}}1} \right)/{\rm{A}}0} \right)] \times 100\%$$

### DPPH scavenging experiment

Following the manufacturer’s instructions, we used the DPPH free radical scavenging capacity assay kit (BC4755, SOLARBIO) to assess the scavenging capacity of ginsenoside Rb1 and RBCQDs on free radicals. Ginsenoside Rb1 and RBCQDs were briefly mixed with the DPPH solution and then incubated at room temperature for 30 min. The absorbance of the solution was measured at 517 nm (A1). Absorbance values were obtained by replacing the sample with PBS (A0). The DPPH radical scavenging rate (%)= [(A0-A1)/(A0)] × 100%.

### Iron ion chelation experiment

To assess the iron chelation effect of ginsenoside Rb1 and RBCQDs, the fluorescence quenching degree and UV absorption spectral changes were compared before and after mixing with iron ions (Fe^2+^ or Fe^3+^). Specifically, Fe^2+^ or Fe^3+^ in the 0–1 mM range was added to the RBCQDs solution (3 mg/ml) and thoroughly mixed. The fluorescence emission spectra were measured using a fluorescence spectrometer at an excitation wavelength of 435 nm. For ginsenoside Rb1 and RBCQDs, solutions with 100, 200, 300, and 400 μg/ml were added to 1 mM Fe^2+^ or Fe^3+^ solutions. UV spectra of ginsenoside Rb1 and RBCQDs in the presence of iron ions were recorded using a multifunctional microplate reader, and the changes in the UV spectra were compared.

### Cell culture

HT22 mouse hippocampal neuronal cells were obtained from the American Type Culture Collection (ATCC) and cultured in Dulbecco’s Modified Eagle Medium (DMEM) supplemented with 10% fetal bovine serum, 100 U/ml penicillin, and 100 µg/ml streptomycin. All cells were maintained in an incubator at 37 °C with 5% CO2.

### Cell viability assay

HT22 cells were randomly divided into four groups and seeded into a 96-well plate: Control group, RBCQDs group, Hemin group, and Hemin + RBCQDs group. Cells in the Control, RBCQDs, Hemin + CSF, and Hemin + RBCQDs groups were pretreated with 0, 25, 0, and 25 µg/ml of RBCQDs respectively for 12 h, followed by exposure to 0, 0, 100, and 100 µM of Hemin respectively for 24 h. Each well was then treated with 10 µl of CCK-8 solution. After a 4-hour incubation at 37 °C, the absorbance at 450 nm was measured.

### Flow cytometry measurement of ROS

HT22 cells were seeded in a 6-well plate, randomized into different groups described above, and subjected to RBCQDs pretreatment and Hemin exposure. The intracellular ROS levels in other groups were detected using the DCFH-DA fluorescent probe. Flow cytometry measured and quantified the fluorescence intensity in the FITC channel of HT22 cells.

### Non-heme iron ion detection experiment

Brain tissue samples were collected on the 3 days. In brief, according to the manufacturer’s instructions, the Iron Assay Kit (ab83366, Abcam) was used to detect the iron ion content in tissues.

### Animal experiments

All mouse experiments were conducted following the ’Guidelines for the Care and Use of Laboratory Animals’ and approved by the Animal Ethics Committee of Nanchang University (Protocol Number: NCULAE-2,023,061,0001). Male C57BL/6J mice (8–10 weeks old) were used and obtained from Nanjing Kyurius Animal Co., Ltd. All mice were housed under standard conditions with a 12 h light/dark cycle (lights on at 6:00 AM, off at 6:00 PM), a temperature of 22 ± 1 °C, humidity > 30%, and ad libitum access to food and water.

### ICH model and RBCQDs treatment

C57BL/6J mice (23-25 g) were induced with 2.5% isoflurane and maintained under anesthesia with 1.5% using a stereotaxic apparatus (68,537, RWD). Collagenase IV (67 U/ml, Sigma-Aldrich) was injected into the striatum at a dose of 0.04 µl/g (coordinates: 0.4 mm anterior to bregma, 2.2 mm right and 3.1 mm deep). A heating pad maintained the body temperature at 37 °C throughout the surgery. After the surgery, mice were provided ample food and water upon anesthesia recovery.

Mice were randomly divided into four groups: (1) Sham group: mice received intrathecal injection of 5 µl artificial cerebrospinal fluid (CSF,0.2 µl/g); (2) RBCQDs group: mice received intrathecal injection of RBCQDs (3 mg/ml) at a dose of 0.2 µl/g; (3) ICH + CSF group: mice underwent collagenase injection into the striatum and received an intrathecal injection of artificial CSF at a dose of 0.2 µl/g; (4) ICH + RBCQDs: mice underwent collagenase injection into the striatum and received an intrathecal injection of RBCQDs (3 mg/ml) at a dose of 0.2 µl/g.

### Behavioral assessment

Before modeling and on days 1, 3, and 7 post-ICH, forelimb grip strength, left hindlimb pain threshold, and mNSS were assessed in mice (*n* = 6/group).

#### Frozen sectioning and HE staining

Tissues were fixed with paraformaldehyde for 48 h, dehydrated in 20% and 30% sucrose gradient, and sectioned into 15 μm slices using a cryostat (CM1952, Leica). For HE staining, tissues were stained with hematoxylin and eosin following the manufacturer’s instructions. Slices were imaged using an optical microscope (DMi8, Leica).

#### Observation of drug distribution

Observation of drug distribution was included in in vivo and ex vivo assessments. In vivo observation was conducted using an imaging system (IVIS, PerkinElmer) at different times: before intrathecal injection of Cy5-RBCQDs and 5 min, 30 min, and 3 h after injection. Ex vivo observation involved fixing and cryosectioning the brains of mice 30 min and 3 days after intrathecal injection of RBCQDs. The distribution of RBCQDs in the brain was observed using a slide scanner (VS120, Olympus) under an excitation wavelength of 488 nm.

#### Western blot analysis

Brain tissues from the ICH hematoma side were homogenized on ice. Purified proteins were quantified using the BCA protein assay kit. After SDS-PAGE gel electrophoresis and transfer to a PVDF membrane, the membrane was incubated with primary antibodies against MDA (ab27642), Cleaved caspase-3 (ab214430), and GAPDH (ab9485). Following secondary antibody incubation, chemiluminescence imaging was performed using the Tanon 5200 chemiluminescence system.

#### Immunofluorescence staining

As mentioned earlier, brain tissues were sectioned into 15 μm thickness frozen slices. The slices were incubated overnight with primary antibodies against MDA (ab27644) and Cleaved caspase-3 (ab214430). Subsequently, FITC and TRITC fluorescent secondary antibodies were used for 4 h. DAPI (ab285390) and Nissl (N21479) were used for neuronal staining.

#### Detection of brain tissue ROS levels

According to the manufacturer’s instructions, brain tissue ROS levels were measured using the Reactive Oxygen Species Assay Kit (C1300, APPLYGEN). In brief, brain tissues were prepared into single cell suspensions, and the DCFH-DA fluorescent probe was added to the cell suspension at a concentration of 10 µM. After incubating the cells at 37 °C for 30 min, centrifugation was performed at 1000 g for 5 min to collect cell precipitates. The precipitates were washed twice with PBS resuspended in PBS, and the fluorescence intensity was measured using a fluorescence spectrophotometer at an excitation wavelength of 488 nm.

#### Brain water content measurement

The brain water content was measured using the wet-dry weight method [35]. The calculation formula was [(wet weight - dry weight)/(wet weight)] × 100%.

#### Hematoma volume measurement

Following the protocol described in the literature [36], mouse brains were consecutively coronally sliced to quantify hematoma volume. The steps involved fixing the mouse brain in paraformaldehyde for 48 h and using a vibrating microtome (VT1200 S, LEICA) to cut the brain into 1 mm slices in the coronal plane. The hematoma volume was assessed by measuring the size of the hematoma in the brain using ImageJ software: hematoma volume = sum of hematoma areas × slice thickness.

#### Mouse forelimb traction test

The forelimb traction force recovery of mice was measured according to a published protocol [37]. Mice were placed steadily on a measuring board, and when the mice were calm, their tails were slowly pulled backward until they released their forelimbs. The instrument recorded the numerical value of the forelimb grip strength.

#### Mouse Hind paw pain threshold

The recovery of pain perception in mouse hind limbs was measured according to a published protocol [38]. Mice were placed in a measuring cage, and after the mice adapted to the surrounding environment, a fine needle was vertically placed on the sole of the mouse’s left hind paw. The needle was slowly lifted until the mouse lifted its left hind paw off the needle, and the maximum force applied by the needle during this process was read on the instrument as the mouse’s back paw pain threshold.

#### Modified neurological severity score (mNSS)

The recovery of mouse neurological function was comprehensively assessed using the mNSS [39]. The mNSS score comprises 10 tasks, including assessments of the mouse’s motor function (muscle status, abnormal movements), sensory function (visual, tactile, and proprioceptive sensation), balance, and reflexes. The scores range from 0 to 18, with higher scores indicating more significant neurological damage.

#### Cerebral meningeal blood Flow Measurement

Following the described procedure, the scalp was incised to expose the skull after anesthetizing the mice. The laser speckle blood flow imaging system (LSI, RWD) was used to measure the cerebral meningeal blood flow on the mouse brain surface. Each measurement was recorded for 10 s, and the average value was recorded.

### Statistical analysis

ImageJ software was used to measure brain slice hematoma area and RBCQDs’ fluorescent distribution in brain tissues. Statistical analysis of experimental data was performed using GraphPad Prism 8 software. All data are presented as mean ± standard deviation. Independent sample t-tests or paired sample t-tests were conducted for comparisons between two groups, and one-way analysis of variance (ANOVA) with Turkey’s post hoc test was employed for comparisons among multiple groups. Two-way ANOVA with Turkey’s post hoc test was used to assess the effects of two factors. A significance level of *P* < 0.05 was considered statistically significant, and * represents *p* < 0.05.

### Electronic supplementary material

Below is the link to the electronic supplementary material.

Supplementary Material 1: Fig. S1 Characterization of ginsenoside Rb1 and RBCQDs. (**a**) HRTEM image of RBCQDs. (**b**) XPS survey spectrum of RBCQDs. XPS survey spectrum (**c**), C1s peak (**d**) and O1s peak (**e**) of ginsenoside Rb1. Fig. S2 Chelation of iron ions by ginsenoside Rb1 and RBCQDs. Images before and after mixing ginsenoside Rb1 with Fe^2+^ (**a**) and Fe^3+^ (**b**). Bright-field and 365 nm UV images after mixing CQDs with different concentrations of Fe^2+^ (**c**) and Fe^3+^ (**d**). Fig. S3 Stern-Volmer plots of Fe^2+^ and Fe^3+^ on RBCQDs. Fig. S4 UV spectra of RBCQDs at different concentrations. Fig. S5 UV spectra of ginsenoside Rb1 binding to iron ions. UV spectra of ginsenoside Rb1 (**a**), ginsenoside Rb1 binding to Fe^2+^ (**b**), and ginsenoside Rb1 binding to Fe^3+^ (**c**). Fig. S6 The content of RBCQDs in the brain. The content (**a**) and relative fluorescence intensity (**b**) of RBCQDs within the brain at 30 min and 3 days post-intrathecal injection. Fig. S7 HE staining images of major organs in Sham and RBCQDs groups of mice. Fig. S8 Weight loss and recovery of mice before ICH, 1 day after ICH, 3 days after ICH, and 7 days after ICH in Sham vs. RBCQDs and ICH + CSF vs. ICH + RBCQDs groups

## Data Availability

The data supporting this study will be available upon request.

## References

[1] Sheth KN (2022). Spontaneous intracerebral hemorrhage. N Engl J Med.

[2] Tu WJ, Wang LD (2023). China stroke surveillance report 2021. Military Med Res.

[3] de Oliveira Manoel AL, Goffi A, Zampieri FG, Turkel-Parrella D, Duggal A, Marotta TR (2016). The critical care management of spontaneous intracranial hemorrhage: a contemporary review. Crit Care.

[4] Farr AC, Xiong MP (2020). Challenges and opportunities of deferoxamine delivery for treatment of Alzheimer’s disease, Parkinson’s disease, and intracerebral hemorrhage. Mol Pharm.

[5] Hirvonen T, Virtamo J, Korhonen P, Albanes D, Pietinen P (2000). Intake of flavonoids, carotenoids, vitamins C and E, and risk of stroke in male smokers. Stroke.

[6] Liu Y, Zhu W, Ni D, Zhou Z, Gu Jh, Zhang W (2020). Alpha lipoic acid antagonizes cytotoxicity of cobalt nanoparticles by inhibiting ferroptosis-like cell death. J Nanobiotechnol.

[7] Wu J, Hecker JG, Chiamvimonvat N (2009). Antioxidant enzyme gene transfer for ischemic diseases. Adv Drug Deliv Rev.

[8] Cai R, Xiao L, Liu M, Du F, Wang Z. Recent advances in functional carbon quantum dots for antitumour. Int J Nanomed. 2021;p. 7195–229. 10.2147/IJN.S334012 PMC8550800 34720582

[9] Ye P, Li L, Qi X, Chi M, Liu J, Xie M (2023). Macrophage membrane-encapsulated nitrogen-doped carbon quantum dot nanosystem for targeted treatment of Alzheimer’s disease: regulating metal ion homeostasis and photothermal removal of β-amyloid. J Colloid Interface Sci.

[10] Luo W, Wang Y, Lin F, Liu Y, Gu R, Liu W et al. Selenium-doped carbon quantum dots efficiently ameliorate secondary spinal cord injury via scavenging reactive oxygen species. Int J Nanomed. 2020;p. 10113–25. 10.2147/IJN.S282985 PMC7754097 33363370

[11] Gong L, Yin J, Zhang Y, Huang R, Lou Y, Jiang H (2022). Neuroprotective mechanisms of ginsenoside Rb1 in central nervous system diseases. Front Pharmacol.

[12] Liu Y, Zhu H, Zhou W, Ye Q (2020). Anti-inflammatory and anti-gouty-arthritic effect of free Ginsenoside Rb1 and nano Ginsenoside Rb1 against MSU induced gouty arthritis in experimental animals. Chemico-Biol Interact.

[13] Lin CJ, Chang L, Chu HW, Lin HJ, Chang PC, Wang RY (2019). High amplification of the antiviral activity of curcumin through transformation into carbon quantum dots. Small.

[14] Guo Y, Zhang L, Zhang S, Yang Y, Chen X, Zhang M (2015). Fluorescent carbon nanoparticles for the fluorescent detection of metal ions. Biosens Bioelectron.

[15] Veloso AD, Ferraria AM, Botelho do Rego AM, Tavares PB, Valentão P, Pereira DD (2019). Hydrophilic carbon nanomaterials: characterisation by physical, chemical, and biological assays. ChemMedChem.

[16] Limosani F, Bauer EM, Cecchetti D, Biagioni S, Orlando V, Pizzoferrato R (2021). Top-down N-doped carbon quantum dots for multiple purposes: heavy metal detection and intracellular fluorescence. Nanomaterials.

[17] Alkian I, Sutanto H (2022). Quantum yield optimization of carbon dots using response surface methodology and its application as control of Fe^3+^ ion levels in drinking water. Mater Res Express.

[18] Ma YX, Xing D, Shao WJ, Du XY, La PQ (2017). Preparation of polyamidoamine dendrimers functionalized magnetic graphene oxide for the adsorption of hg (II) in aqueous solution. J Colloid Interface Sci.

[19] Chen J, Li M, Huo X, Li Z, Qu D, Sha J (2023). A novel process for one-step separation and cyclodextrin inclusion of Ginsenoside Rg5 from Ginseng Stem–Leaf Saponins (GSLS): Preparation, characterization, and Evaluation of Storage Stability and Bioactivity. Foods.

[20] Lu W, Qin X, Liu S, Chang G, Zhang Y, Luo Y (2012). Economical, green synthesis of fluorescent carbon nanoparticles and their use as probes for sensitive and selective detection of mercury (II) ions. Anal Chem.

[21] Hou J, Wang W, Zhou T, Wang B, Li H, Ding L (2016). Synthesis and formation mechanistic investigation of nitrogen-doped carbon dots with high quantum yields and yellowish-green fluorescence. Nanoscale.

[22] Yang Y, Wang Q, Li G, Guo W, Yang Z, Liu H (2023). Cysteine-derived chiral Carbon Quantum dots: a fibrinolytic activity Regulator for Plasmin to target the human islet amyloid polypeptide for type 2 diabetes Mellitus. ACS Appl Mater Interfaces.

[23] Sudhakar M, Djurovich PI, Hogen-Esch TE, Thompson ME (2003). Phosphorescence quenching by conjugated polymers. J Am Chem Soc.

[24] Dharmalingam P, Talakatta G, Mitra J, Wang H, Derry PJ, Nilewski LG (2020). Pervasive genomic damage in experimental intracerebral hemorrhage: therapeutic potential of a mechanistic-based carbon nanoparticle. ACS Nano.

[25] Li S, Jiang D, Ehlerding EB, Rosenkrans ZT, Engle JW, Wang Y (2019). Intrathecal administration of nanoclusters for protecting neurons against oxidative stress in cerebral ischemia/reperfusion injury. ACS Nano.

[26] Wang M, Zhou C, Yu L, Kong D, Ma W, Lv B (2022). Upregulation of MDH1 acetylation by HDAC6 inhibition protects against oxidative stress-derived neuronal apoptosis following intracerebral hemorrhage. Cell Mol Life Sci.

[27] Li S, Wang Y, Wu M, Younis MH, Olson AP, Barnhart TE (2022). Spleen-targeted glabridin- loaded nanoparticles regulate polarization of Monocyte/Macrophage (Mo/Mφ) for the treatment of cerebral ischemia-reperfusion Injury. Adv Mater.

[28] Aspelund A, Antila S, Proulx ST, Karlsen TV, Karaman S, Detmar M (2015). A dural lymphatic vascular system that drains brain interstitial fluid and macromolecules. J Exp Med.

[29] Patel TK, Habimana-Griffin L, Gao X, Xu B, Achilefu S, Alitalo K (2019). Dural lymphatics regulate clearance of extracellular tau from the CNS. Mol Neurodegeneration.

[30] Wu H, Dunnett S, Ho YS, Chang RCC (2019). The role of sleep deprivation and circadian rhythm disruption as risk factors of Alzheimer’s disease. Front Neuroendocr.

[31] Ma Q, Ineichen BV, Detmar M, Proulx ST (2017). Outflow of cerebrospinal fluid is predominantly through lymphatic vessels and is reduced in aged mice. Nat Commun.

[32] Wan Y, Holste KG, Hua Y, Keep RF, Xi G (2023). Brain edema formation and therapy after intracerebral hemorrhage. Neurobiol Dis.

[33] Puy L, Parry-Jones AR, Sandset EC, Dowlatshahi D, Ziai W, Cordonnier C (2023). Intracerebral haemorrhage. Nat Reviews Disease Primers.

[34] FEIa X, LIUb Y, Li C, Guo J, SYNTHESIS, FLUORESCENCE PROPERTIES OF A COMPLEX, PROBE BASED ON HYDROXYL QUANTUM DOTS AND CARBAZOLE ABUTMENT STYRYL FLUORESCENCE DYE (2012). Chalcogenide Lett.

[35] Tian Q, Guo Y, Feng S, Liu C, He P, Wang J (2022). Inhibition of CCR2 attenuates neuroinflammation and neuronal apoptosis after subarachnoid hemorrhage through the PI3K/Akt pathway. J Neuroinflamm.

[36] Gautam J, Xu L, Nirwane A, Nguyen B, Yao Y (2020). Loss of mural cell-derived laminin aggravates hemorrhagic brain injury. J Neuroinflamm.

[37] Li X, Chen C, Zhan X, Li B, Zhang Z, Li S (2021). R13 preserves motor performance in SOD1G93A mice by improving mitochondrial function. Theranostics.

[38] Zhou Yq L, Dq C, Sp, Chen N, Sun J, Wang X (2020). Nrf2 activation ameliorates mechanical allodynia in paclitaxel-induced neuropathic pain. Acta Pharmacol Sin.

[39] Xiao L, Zheng H, Li J, Zeng M, He D, Liang J (2022). Targeting NLRP3 inflammasome modulates gut microbiota, attenuates corticospinal tract injury and ameliorates neurobehavioral deficits after intracerebral hemorrhage in mice. Biomed Pharmacother.

