# Correction: Carbon quantum dots of ginsenoside Rb1 for application in a mouse model of intracerebral hemorrhage

**DOI:** 10.1186/s12951-024-02497-2

**Published:** 2024-05-24

**Authors:** Xiaolong Tang, Xinyu Yang, Yamei Yu, Miaojing Wu, Yuanyuan Li, Zhe Zhang, Guangyu Jia, Qi Wang, Wei Tu, Ye Wang, Xingen Zhu, Shiyong Li

**Affiliations:** 1 Department of Neurosurgery, The Second Affiliated Hospital, Jiangxi Medical College, Nanchang University, Nanchang, Jiangxi 330006 China; 2 Institute of Neuroscience, Nanchang University, Nanchang, Jiangxi 330006 China; 3 Department of Neurology, The Second Affiliated Hospital, Jiangxi Medical College, Nanchang University, Nanchang, Jiangxi 330006 China

**Correction: Journal of Nanobiotechnology(2024)22:125**

10.1186/s12951-024-02368-w

Following publication of the original article, details for affiliations of all authors were incorrectly given as:

1. Institute of Neuroscience, Jiangxi Medical College, Nanchang University, Street, Nanchang, Jiangxi 330036, China

2. Department of Neurosurgery, The Second Affiliated Hospital, Jiangxi Medical College, Nanchang University, Street, Nanchang, Jiangxi 330008, China

3. Department of Neurology, The Second Affiliated Hospital, Jiangxi Medical College, Nanchang University, Street, Nanchang, Jiangxi 330008, China

but should have been:

1. Department of Neurosurgery, The Second Affiliated Hospital, Jiangxi Medical College, Nanchang University, Nanchang, Jiangxi, 330006, China

2. Institute of Neuroscience, Nanchang University, Nanchang, Jiangxi, 330006, China

3. Department of Neurology, The Second Affiliated Hospital, Jiangxi Medical College, Nanchang University, Nanchang, Jiangxi, 330006, China

The original article has been corrected.

