# Supplementary material for: Carbon quantum dots of ginsenoside Rb1 for application in a mouse model of intracerebral Hemorrhage
Source: J Nanobiotechnology. 2024 Mar 22;22:125. doi: 10.1186/s12951-024-02368-w (PMC10958843; doi:10.1186/s12951-024-02368-w)
Supplement: Supplementary file 1 — Supplementary Material 1: Fig. S1 Characterization of ginsenoside Rb1 and RBCQDs. (a) HRTEM image of RBCQDs. (b) XPS survey spectrum of RBCQDs. XPS survey spectrum (c), C1s peak (d) and O1s peak (e) of ginsenoside Rb1. Fig. S2 Chelation of iron ions by ginsenoside Rb1 and RBCQDs. Images before and after mixing ginsenoside Rb1 with Fe2+ (a) and Fe3+ (b). Bright-field and 365 nm UV images after mixing CQDs with different concentrations of Fe2+ (c) and Fe3+ (d). Fig. S3 Stern-Volmer plots of Fe2+ and Fe3+ on RBCQDs. Fig. S4 UV spectra of RBCQDs at different concentrations. Fig. S5 UV spectra of ginsenoside Rb1 binding to iron ions. UV spectra of ginsenoside Rb1 (a), ginsenoside Rb1 binding to Fe2+ (b), and ginsenoside Rb1 binding to Fe3+ (c). Fig. S6 The content of RBCQDs in the brain. The content (a) and relative fluorescence intensity (b) of RBCQDs within the brain at 30 min and 3 days post-intrathecal injection. Fig. S7 HE staining images of major organs in Sham and RBCQDs groups of mice. Fig. S8 Weight loss and recovery of mice before ICH, 1 day after ICH, 3 days after ICH, and 7 days after ICH in Sham vs. RBCQDs and ICH + CSF vs. ICH + RBCQDs groups [file 12951_2024_2368_MOESM1_ESM.docx]

Carbon Quantum Dots of Ginsenoside Rb1 for Application in a Mouse Model of Intracerebral Hemorrhage

Xiaolong Tang1,2***†***, Xinyu Yang1,2***†***, Yamei Yu1,3***†***, Miaojing Wu1, 2***†***, Yuanyuan Li1, Zhe Zhang1,2, Guangyu Jia1,2, Qi Wang1,2, Wei Tu1,2*, Ye Wang1,3*, Xingen Zhu1,2*, Shiyong Li1,2*

1*Institute of Neuroscience, Jiangxi Medical College, Nanchang University, Street, Nanchang, Jiangxi, 330036, China.

2Department of Neurosurgery, The Second Aﬀiliated Hospital, Jiangxi Medical College, Nanchang University , Street, Nanchang, Jiangxi,330008, China.

3Department of Neurology, The Second Aﬀiliated Hospital, Jiangxi Medical College, Nanchang University, Street, Nanchang, Jiangxi, 330008, China.

*Corresponding author(s). E-mail(s): sylicg@ncu.edu.cn; ndefy89006@ncu.edu.cn; ndefy08041@ncu.edu.cn; Ndefy06044@ncu.edu.cn;

Contributing authors: 413007210224@email.ncu.edu.cn; 353007210006@email.ncu.edu.cn; 403007220020@email.ncu.edu.cn; ndefy15119@ncu.edu.cn; ndefy21215@ncu.edu.cn; 363007220042@email.ncu.edu.cn; 403007220005@email.ncu.edu.cn; 403007230003@email.ncu.edu.cn;

***†***These authors contributed equally to this work.


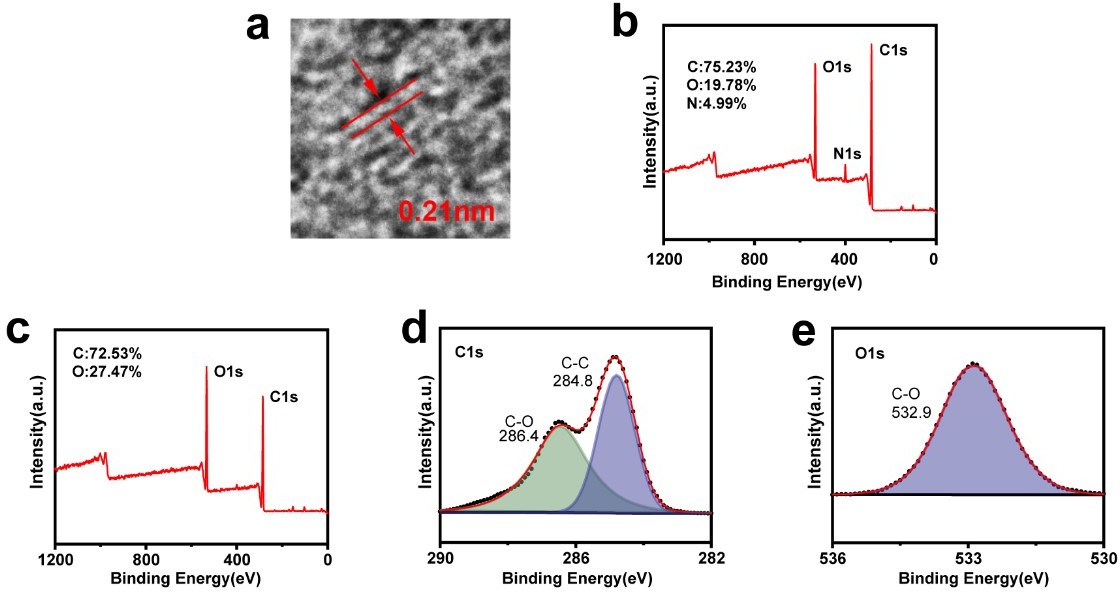


**Fig. S1** Characterization of ginsenoside Rb1 and RBCQDs. (a) HRTEM image of RBCQDs. (b) XPS survey spectrum of RBCQDs. XPS survey spectrum (c), C1s peak (d), and O1s peak (e) of ginsenoside Rb1.


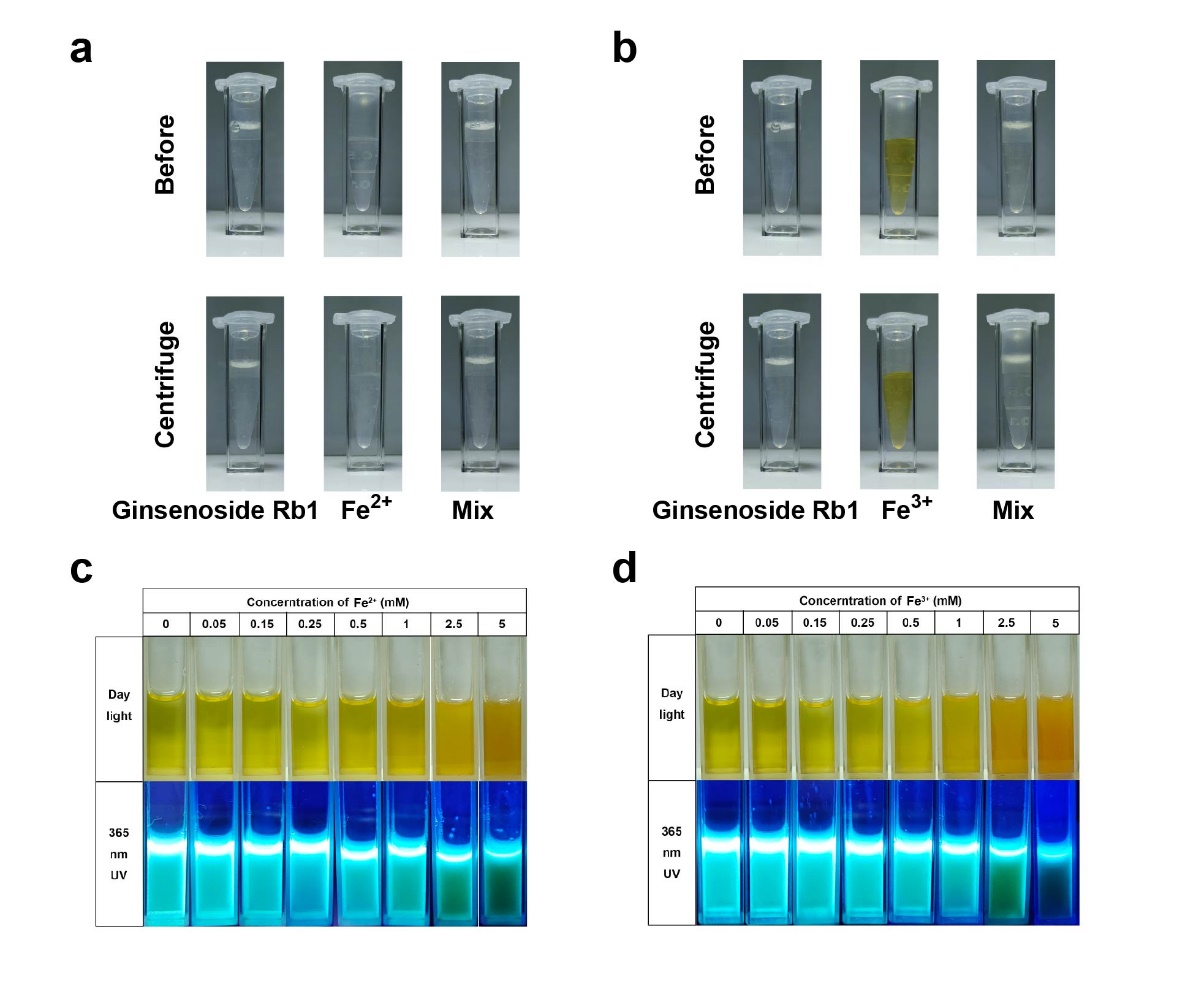


**Fig. S2** Chelation of iron ions by ginsenoside Rb1 and RBCQDs. Images before and after mixing ginsenoside Rb1 with Fe^2+^ (a) and Fe^3+^ (b). Bright-field and 365nm UV images after mixing CQDs with different concentrations of Fe^2+^ (c) and Fe^3+^ (d).


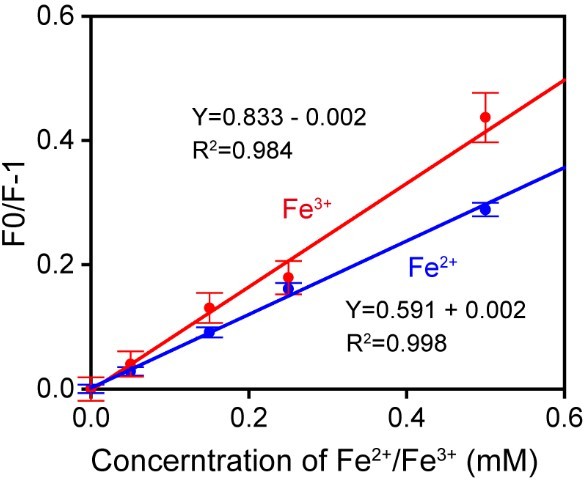


**Fig. S3** Stern-Volmer plots of Fe^2+^ and Fe^3+^ on RBCQDs (n=6/group).


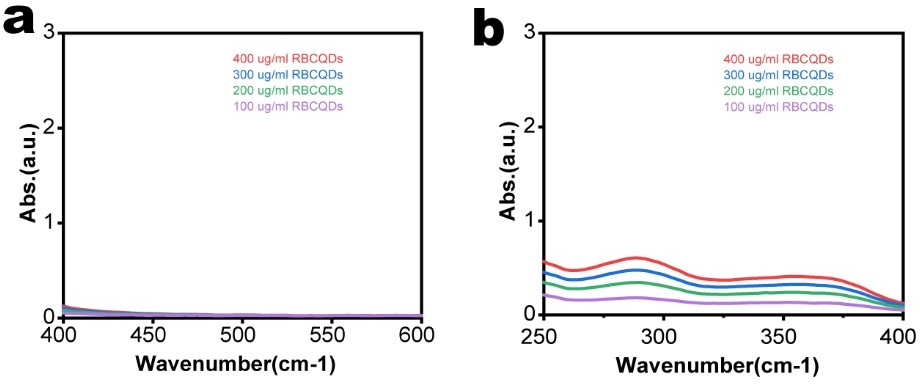


**Fig. S4** UV Spectra of RBCQDs at different concentrations.


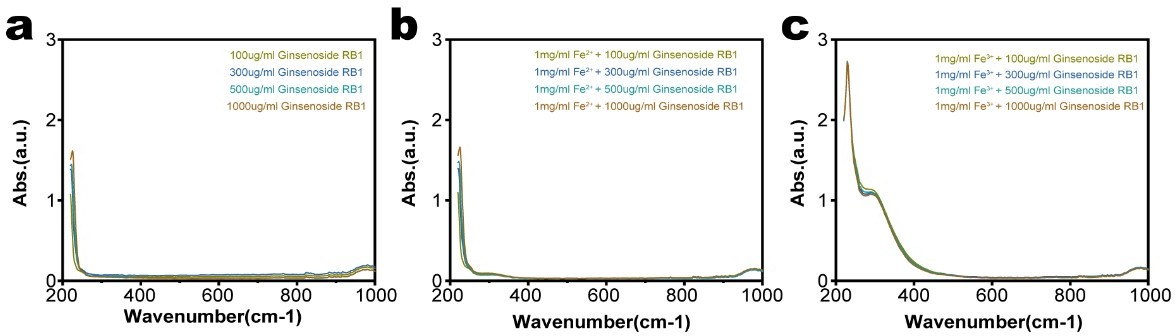


**Fig. S5** UV spectra of ginsenoside Rb1 binding to iron ions. UV spectra of ginsenoside Rb1 (a), ginsenoside Rb1 binding to Fe^2+^ (b), and ginsenoside Rb1 binding to Fe^3+^ (c).


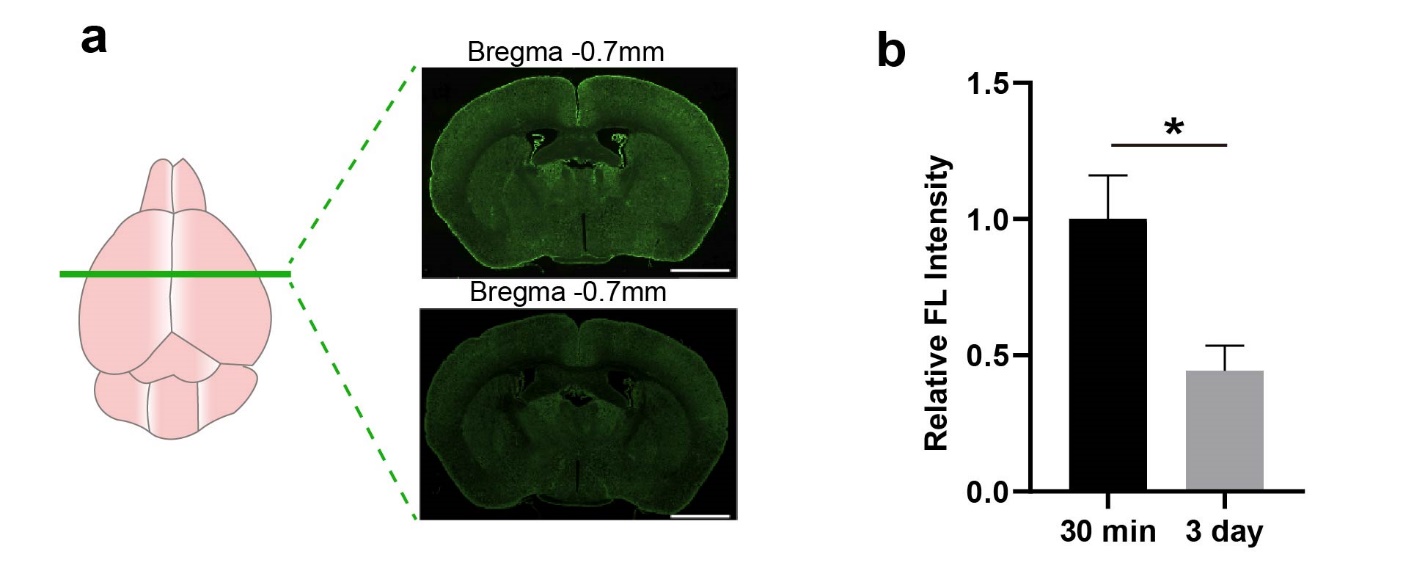


**Fig. S6** The content of RBCQDs in the brain. The content (a) and relative fluorescence intensity (b) of RBCQDs within the brain at 30 minutes and 3 days post-intrathecal injection (n=6/group, unpaired t-test). The scale bar length was 2mm.


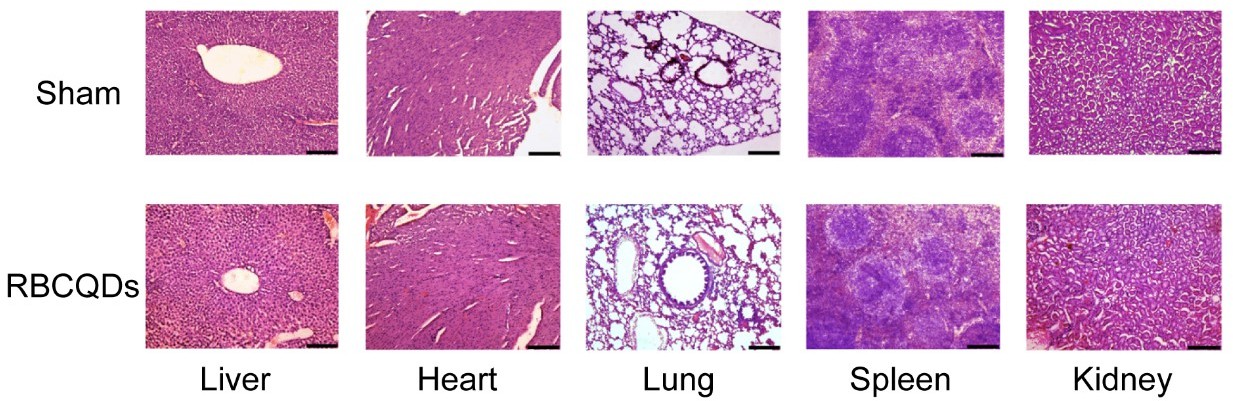


**Fig. S7** HE staining images of major organs in Sham and RBCQDs groups of mice. The scale bar length was 200 µm.


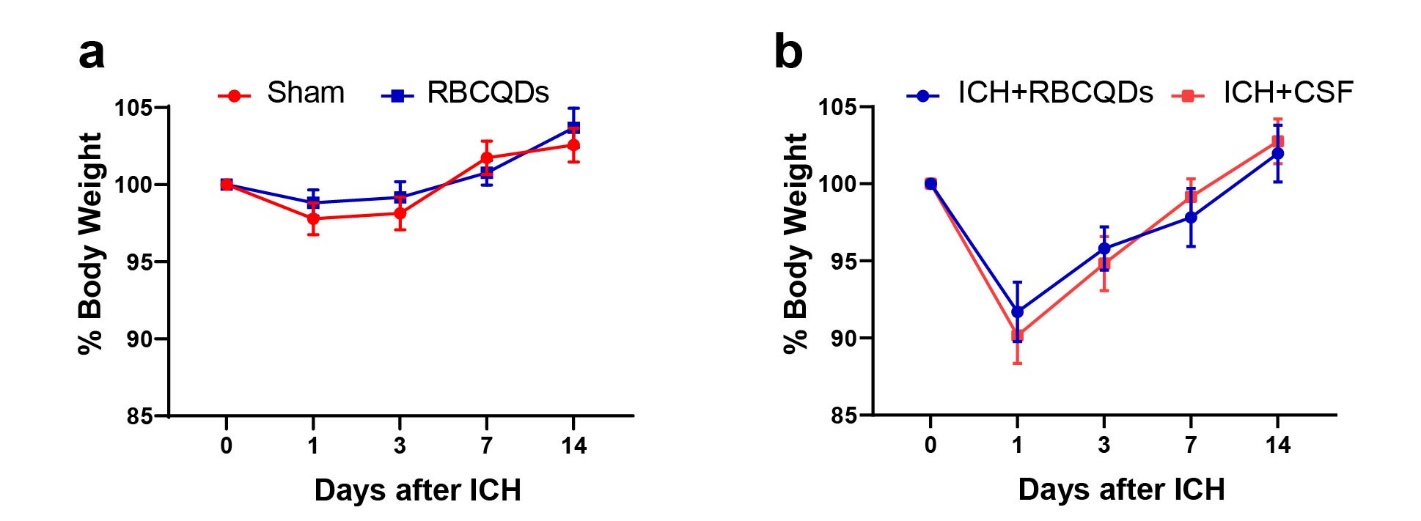


**Fig.** **S8** Weight loss and recovery of mice before ICH, 1 day after ICH, 3 days after ICH, and 7 days after ICH in Sham vs. RBCQDs and ICH + CSF vs. ICH + RBCQDs groups (n=6/group,unpaired t-test).
